# Efficacy and Safety of Drug-Eluting Bead TACE in the Treatment of Primary or Secondary Liver Cancer

**DOI:** 10.1155/2023/5492931

**Published:** 2023-04-26

**Authors:** Jiabing Wang, Haoqian Xu, Ying Wang, Long Feng, Fengming Yi

**Affiliations:** ^1^Department of Oncology, Second Affiliated Hospital of Nanchang University, Nanchang 330006, China; ^2^Jiangxi Key Laboratory of Clinical and Translational Cancer Research, Nanchang 330006, China; ^3^Jiangxi Provincial People's Hospital, Nanchang 330006, China

## Abstract

**Background:**

The drug-eluting beads transarterial chemoembolization (DEB-TACE) has already been used in hepatic malignancies. We aim to evaluate the efficacy and safety of DEB-TACE in treating primary or secondary liver cancer.

**Methods:**

We retrospectively evaluated 59 patients with hepatic malignancies, including 41 patients with primary liver cancer and 18 patients with secondary liver cancer, between September 2016 and February 2019. All patients were treated with DEB-TACE. Objective response rate (ORR) and disease control rate (DCR) were evaluated by mRECIST. The pain was assessed using a numerical rating scale (NRS) where 0 represented no pain, and a score of ten was unbearable. Adverse reactions were assessed according to Common Terminology Criteria for Adverse Events 4.0 (CTCAE4.0).

**Results:**

In the subgroup of primary liver cancer, 3 patients (7.32%) got complete response, 13 patients (31.71%) got partial response, 21 patients (51.22%) experienced stable disease, and 4 patients (9.76%) suffered progressive disease; ORR was 39.02% and DCR was 90.24%. In the subgroup of secondary liver cancer, 0 patients (0%) got complete response, 6 patients (33.33%) got partial response, 11 patients (61.11%) experienced stable disease, and 1 patient (5.56%) suffered progressive disease; ORR was 33.33% and DCR was 94.44%. We did not find any difference when comparing the efficacy between primary and secondary liver cancer (*P*=0.612). The one-year survival rate was 70.73% for primary liver cancer and 61.11% for secondary liver cancer. There was no significant difference between the two groups (*P*=0.52). For the patients with CR or PR, no factor could predict the efficacy of DEB-TACE. The most common treatment-related adverse reactions were short-term liver function disorders. The symptoms included fever (20.34%), abdomen pain (16.95%), and vomiting (5.08%), all patients with adverse reactions got remission after treatment.

**Conclusions:**

DEB-TACE has a promising effect in the treatment of primary or secondary liver cancer. The treatment-related adverse reactions are tolerable.

## 1. Introduction

The incidence and mortality rates of hepatocellular carcinoma (HCC) are rising globally. The five-year survival rate for the early stage of HCC is greater than 70%. However, the median overall survival (mOS) for the advanced stage of HCC is 1–1.5 years [1]. Although tyrosine kinase inhibitors and immune checkpoint inhibitors increase the survival of advanced or unresectable HCC, topical therapy is still an essential treatment in patients with advanced or unresectable HCC (1). Transarterial therapies are one of the vital tools in treating advanced or unresectable HCC, including bland embolization, chemoembolization, and radioembolization. Chemoembolization is considered the most common method, including conventional transarterial chemoembolization (c-TACE) and drug-eluting bead chemoembolization (DEB-TACE). However, which one is superior is controversial [2].

Liver metastasis is one of the most common sites of malignant tumors; colorectal cancer (CRC) liver metastasis, and gastric cancer (GC) liver metastasis was concentrated in systematic treatment and conversion therapy [3, 4]. However, transcatheter-direct and percutaneous locoregional therapies have evolved as major therapy modalities for unresectable metastatic disease. These locoregional treatments have increased tumor response, and improved disease-free survival (DFS) and OS in a broad range of metastatic diseases [5]. DEB-TACE is considered one of the essential approaches in liver metastasis patients who fail standard treatment regimens. A study concluded that regorafenib combined with DEB-TACE is superior to regorafenib monotherapy when considering the response, progression-free survival (PFS), and OS [6]. DEB-TACE and c-TACE demonstrated safety, feasibility, and short-term efficacy in treating gastric cancer liver metastasis [7].

Herein, we aim to explore the efficacy and safety of DEB-TACE in treating primary or secondary liver cancer to verify the DEB-TACE in hepatic malignancies for future clinical treatment.

## 2. Methods

### 2.1. General Information

We evaluated the outcomes of patients with hepatic malignancies who could not receive curative surgery or transplantation in the Second Affiliated Hospital of Nanchang University from September 2016 to February 2019. 59 patients with hepatic malignancies (41 primary liver cancer and 18 secondary liver cancer) were evaluated. The inclusion criteria for the research were [1] pathology or radiology confirmed primary liver cancer; radiology should be according to American Association for the Study of Liver Diseases (AASLD) criteria. Pathology confirmed secondary liver cancer; [2] ages between 18 and 80; [3] Eastern Cooperative Oncology Group (ECOG) physical status score restricted to 0–2; [4] Child–Pugh score should be A or B; [5] informed consent has been signed. Exclusion criteria included [1] severe liver and kidney dysfunction and serious underlying diseases; [2] coagulation dysfunction that cannot be corrected; [3] complete obstruction of main portal vein carcinoma thrombus without compensated collateral circulation; [4] hepatic arterio-venous fistula; [5] expected life-long lower than three months; [6] tumor volume should not exceed 70% of liver volume. [7] Baseline lesions cannot be measured by the modified response evaluation criteria in solid tumors (mRECIST). This study was approved by the Ethics Committee of the Second Affiliated Hospital of Nanchang University.

### 2.2. Treatment

The method for the use of drug-loaded microspheres are as follows: 0.9% sodium chloride solution with a 20 ml syringe was added to the bottle of CalliSpheres (diameter: 100–300 *μ*m, Suzhou HengruiGalisheng Biomedical Technology Co., Ltd., Suzhou, China), erected the syringe upward until the microspheres were precipitated, and discharged the supernatant completely. Dissolved the chemotherapeutic agent with a 5 ml syringe, and then connected it with the 20 ml syringe equipped with a microsphere using a T-junction. Mixed the chemotherapeutic drug and the microsphere in the 20 ml syringe, then shook the 20 ml syringe every 5 minutes. After 30 minutes of chemotherapeutic drug adsorption for the microsphere, the final solution was mixed with the contrast agent 1 : 1 for use.

The treatment process of drug-eluting bead TACE is described as follows: all procedures for the embolic intervention were performed on a digital subtraction angiography (DSA) machine(Allura Xper FD20, Philips, Netherlands). Seldinger technique was used to perform femoral artery puncture. 5F RH catheter or Yashiro catheter and 2.8F microcatheter were superselected to the tumor blood supply artery, and the 100−300 *μ*m chemotherapeutic agents loaded CalliSpheres were slowly injected into the artery supply of the tumor. Using pulse injection, the injection speed was 1-2 ml/min; finally, we could find the complete embolization for the blood supply of tumors.

### 2.3. Assessment

The patients were assessed by computed tomography after the treatment for two months. And the efficacy was evaluated according to mRECIST. Objective response rate (ORR) was defined as the percentage of patients' treatment results that reached complete response (CR) or partial response (PR). Disease control rate (DCR) was defined as the proportion of patients with CR, PR, or stable disease (SD). Treatment response was assessed by the radiology department of our hospital independently. Moreover, we took follow-ups every two months. OS was defined as the length of time from the start of DEB-TACE for liver cancer during the 12-month follow-up. Adverse reactions were recorded during intervention and posttreatment, and general condition and laboratory results were recorded. The pain was evaluated using a numerical rating scale (NRS), where 0 was no pain and ten was unbearable. Adverse reactions were assessed according to Common Terminology Criteria for Adverse Events 4.0 (CTCAE4.0).

### 2.4. Statistical Analysis

We used SPSS 23.0 to process and analyze the statistics. We did the test for the normality of variables. The nonparametric test was used for values with nonnormal distribution or uneven variance. The *t*-test was used when variables meet normal distribution. Chi-square test or Fisher's exact counting method was used for statistical analysis. We used the log-rank method to do a statistical test of the survival curve. *P* < 0.05 indicated a significant difference.

## 3. Results

### 3.1. Patient Characteristics

A total of 59 patients with malignant hepatic tumors were included in this study, including 41 primary liver cancer and 18 secondary liver cancer (5 gastric cancer liver metastasis, 8 colorectal cancer liver metastasis, and 5 other parts originated cancer liver metastasis). The patients' characteristics are shown in Tables 1 and 2, including age, sex, history of hepatitis, alcohol history, portal vein thrombosis, and liver function.

### 3.2. Efficacy

**Figure 1:**
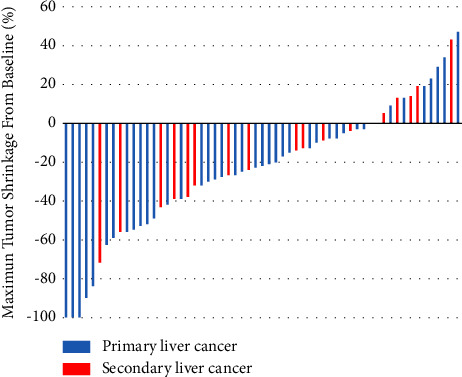
The tumor shrinkage from baseline.

**Figure 2:**
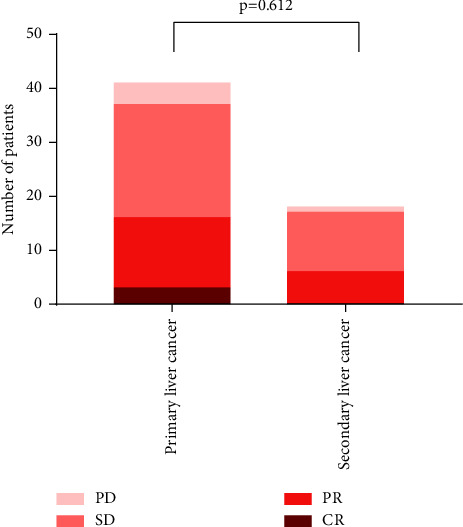
The comparison of effectiveness between primary liver cancer and secondary liver cancer treated with DEB-TACE.

The assessment of treatment effectiveness was according to mRECIST. When patients were defined as CR, PR, or SD, we considered them effective. Patients who were evaluated as PD were deemed noneffective. In the subgroup of primary liver cancer, 3 patients (7.32%) got CR, 13 patients (31.71%) got PR, 21 patients (51.22%) experienced SD, and 4 patients (9.76%) suffered PD; ORR was 39.02% and DCR was 90.24%. In the subgroup of secondary liver cancer, 0 patients (0%) got CR, 6 patients (33.33%) got PR, 11 patients (61.11%) experienced SD, and 1 patient (5.56%) suffered PD; ORR was 33.33% and DCR was 94.44%. The results are presented in Figure 1 and Table 3. Furthermore, we did not find any difference when comparing the efficacy between primary and secondary liver cancer (*P*=0.612, Figure 2).

**Figure 3:**
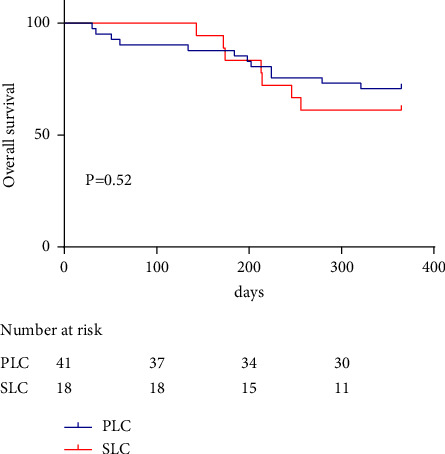
Overall survival of patients with primary liver cancer and secondary liver cancer treated with DEB-TACE. PLC: primary liver cancer; SLC: secondary liver cancer.

The mOS was not reached after a median follow-up of 12 months. The one-year survival rate was 70.73% for primary liver cancer and 61.11% for secondary liver cancer. There was no significant difference between the two groups (*P*=0.52, Figure 3).

### 3.3. Univariate Analysis of Factors Related to Response

For the patients with CR or PR, we tried to get the factors that could predict the efficacy of DEB-TACE. In univariate analysis, there were no significant differences in effectiveness in gender, tumor diameter, history of ablation, or history of TKI or c-TACE (*P* > 0.05). The results are shown in Table 4.

### 3.4. Safety

As for laboratory tests, glutamic-pyruvic transaminase (ALT), glutamic oxalacetic transaminase (AST), and total bilirubin (TB) increased one week after treatment (*P*=0.007, *P*=0.128, and *P*=0.001, respectively), but they all returned to pretreatment level 1–3 months after DEB-TACE (*P*=0.282, *P*=0.703, and *P*=0.451, respectively). The alpha-fetoprotein (AFP) showed no significance between before and after treatment for primary liver cancer patients (*P*=0.295). Both carcinoembryonic antigen (CEA) and carbohydrate antigen-199 (CA-199) did not present any change after intervention (*P*=0.971 and *P*=0.264, respectively).

The most common treatment-related adverse reactions were fever (20.34%), abdomen pain (16.95%), and vomiting (5.08%); all patients got remission after treatment.

## 4. Discussion

TACE is a palliative option for unresectable HCC. Chemotherapeutic drugs are emulsified in ethiodized oil and delivered to hepatic arteries parasitized by tumors in case. DEB-TACE is performed with drug-eluting embolic microspheres and delivered to target arteries. c-TACE and DEB-TACE are controversial when considering the better option in unresectable HCC [8, 9]. A recent meta-analysis including 30 studies compared with c-TACE and DEB-TACE concluded that patients with DEB-TACE might get a better result when considering complete response rate, disease control rate, and 3-year survival rate. Still, the safety did not significantly differ between c-TACE and DEB-TACE [8]. A study was presented to evaluate the prognostic factors for TACE in hepatitis B related HCC and conclude that DEB-TACE is an independent better factor for those patients mentioned [10]. Moreover, the Chinese expert consensus indicated that DEB-TACE has a more favorable response rate and better survival time in HCC [11]. Apart from c-TACE and DEB-TACE, transarterial radioembolization (TARE) was also considered to be effective in treating intermediate-advanced HCC [12]. Taken together, DEB-TACE is a promising option for patients with HCC.

To date, at least five types of commercially available DEB have been developed, including DC Bead, HepaSphere, Tandem, Lifepearl, and CalliSphere. Doxorubicin is considered the standard chemotherapeutic agent used for DEB-TACE in HCC. A study compared the difference between HepaSpheres and CalliSpheres in HCC and found that CalliSpheres TACE was superior in short-term efficacy and similar in long-term efficacy. Moreover, the safety was the same with HepaSpheres [13]. We herein using CalliSpheres as the drug-eluting embolic microspheres in primary liver cancer and secondary liver cancer; in the subgroup of primary liver cancer, three patients (7.32%) got CR, 13 patients (31.71%) got PR, 21 patients (51.22%) experienced SD, 4 patients (9.76%) suffered PD; ORR was 39.02% and DCR was 90.24%. The results were like the studies concluded in the meta-analysis [8]. The one-year survival rate was 70.73% for primary liver cancer, which might be related to the selection of patients with limited tumor numbers. The efficacy of DEB-TACE with CalliSpheres was comparable with other studies that treated HCC patients with different DEB.

In the subgroup of metastatic liver cancer, 0 patients (0%) got CR, 6 patients (33.33%) got PR, 11 patients (61.11%) experienced SD, and 1 patient (5.56%) suffered PD; ORR was 33.33% and DCR was 94.44%. A study collected 42 colorectal cancer liver metastases (CRLM) patients treated with irinotecan-eluting beads TACE (DEBIRI-TACE) by CalliSpheres microspheres. The result also demonstrated high ORR and OS without grade 3 or grade 4 adverse events [14]. Another study aimed to investigate the efficacy of DEBIRI-TACE accompanied with an arterial infusion of oxaliplatin plus  raltitrexed, and found that one-month ORR could reach up to 78.3% [15]. The efficacy of DEB-TACE in patients with gastric cancer liver metastases was also confirmed. DEB-TACE caused fewer incidences of nausea and vomiting but more fever rates than c-TACE [7]. The evaluation of the efficacy and safety of DEB-TACE with doxorubicin-loaded in unresectable intrahepatic cholangiocarcinoma (ICC) was also presented. Eighty-eight patients with unresectable ICC were treated with CalliSpheres microspheres. The ORR was 65.9%, and adverse events were tolerable [16]. A systematic analysis recruited 23 study cohorts and 1091 patients and found that the ORR for c-TACE and DEB-TACE was 29.4% and 51.2%, respectively [17]. An initial study included 14 patients with 39 liver metastases who were treated with DEB-TACE and demonstrated that the ORR was 71.4% at three months [18]. These results demonstrated that the ORR was higher than our study, and the reason might be related to the patients with metastatic liver cancer that were selected in our study were mostly treated with more than first-line or second-line chemotherapy, although we need more patients to confirm them. However, the results in the previous studies and our study indicated the efficiency of DEB-TACE in secondary liver cancer.

As for complications of DEB-TACE, the liver abscess was considered a severe complication after the DEB-TACE; a retrospective analysis demonstrated the incidence of the liver abscess was 8.76% per patient with DEB-TACE and found that a larger maximum tumor diameter, grade one artery occlusion, and systemic chemotherapy recently might be correlated with liver abscess formation [19]. A study tried to select the optimal microparticle size of DEB-TACE and found out that medium-sized (300–500 *μ*m) CalliSpheres microspheres had similar ORR but less grade three liver toxicity and liver abscess when compared with DEB-TACE with small-sized (100−300 *μ*m) CalliSpheres [20]. Other common adverse events were nausea/vomiting, abdominal pain, and transient elevation of liver transaminase [16]. In our research, ALT, AST, and TB increased one week after treatment, but they all returned to pretreatment level 1–3 months after DEB-TACE. The most common treatment-related adverse reactions were fever (20.34%), pain (16.95%), and vomiting (5.08%); all patients got remission after treatment. No liver abscess was observed in our study. The common adverse reactions are similar to the studies before, but no liver abscess was observed which might be related to smaller tumor diameter, and prophylactic antibiotics were used in our study.

When we considered factors correlated with efficacy, gender, tumor diameter, history of ablation, and history of TKI or c-TACE did not show any relationship between response to DEB-TACE in our study. In a previous study, c-TACE treatment history exhibited independent factors for patients treated with DEB-TACE; DEB-TACE displayed a higher ORR, PFS, and OS when compared with c-TACE in patients with c-TACE history [21]. Another research included 81 patients with advanced-stage HCC treated with DEB-TACE and found out that the number of patients in 100–300 *μ*m group was higher than those in the 300–500 *μ*m group when compared with ORR, PFS, and OS, but the complications were similar [22]. A review recommended the selection of small-sized DEB based on experience, but the presence of portal vein thrombosis and intrahepatic shunts are the contraindications for smaller DEBs [23]. A study demonstrated that the lymphocyte-to-monocyte ratio (LMR) and tumor size were the predictors for OS when considering locoregional treatment for colorectal liver metastasis. LMR greater than 3.96% has significantly longer OS and time to recurrence than the ones below [24]. However, another interesting study showed that >46% AST and >52% ALT increases when compared to the pretreatment value were significantly correlated with ORR [25]. No factors related to the efficacy of DEB-TACE in liver cancer in our study might be correlated with limited cases recruited.

However, there are limitations in our study; the combination of DEB-TACE and other therapeutic tools seems better than DEB-TACE monotherapy. A retrospective propensity-score matched study recruited 174 HCC patients classified as DEB-TACE plus apatinib (D-apatinib) and c-TACE plus apatinib (c-apatinib). After propensity-score matching analysis, D-apatinib showed better efficacy when compared with OS and PFS [26]. Regorafenib plus DEB-TACE was a better treatment than regorafenib monotherapy regarding treatment response, PFS, and OS [6]. Retrospective research evaluated the treatment effectiveness of DEB-TACE together with oxaliplatin plus fluorouracil and leucovorin (FOLFOX)-based hepatic arterial infusion chemotherapy (D-TACE-HAIC) in patients with unresectable HCC. The patients who underwent D-TACE-HAIC have a longer OS (median, 19.0 vs. 14.0 months) than those treated with DEB-TACE. However, the safety data were similar in the two groups [27]. Patients with HCC treated with DEB-TACE and microwave ablation (MWA) demonstrated higher ORR and DCR when compared with MWA treatment alone [28]. In the future, we will try to explore the combination in the treatment of liver cancers. Moreover, there are limitations of our study when considering the novelty; for instance, two recent studies demonstrated the efficacy and safety of CalliSpheres DEB-TACE in HCC, and the results were comparable with our study [29, 30]. In addition, there were limited cases in our study, which should be improved in the future.

## 5. Conclusions

In conclusion, DEB-TACE is effective in primary liver cancer and secondary liver cancer, and the complications were tolerable. No factors were correlated with the response to DEB-TACE. DEB-TACE with other therapeutic tools should be explored to get a higher response rate and more prolonged survival.

## Figures and Tables

**Table 1:** Clinical information of unresectable primary liver cancer patients (*n* = 41).

Characteristics	Statistics
Sex (male/female)	37/4
Age (≥65 years old/<65 years old)	20/21
Medical history	
Hepatitis (yes/no)	36/5
Alcohol (yes/no)	3/38
Liver cirrhosis (yes/no)	26/15
Tumor diameter (≥50 mm/<50 mm)	25/16
Tumor number	2.00 ± 1.70
Portal vein invasion (yes/no)	5/36
ECOG (0/1/2)	2/35/4
Child–Pugh (A/B)	38/3
BCLC (A/B/C)	16/18/7
Treatment history	
Conventional TACE (yes/no)	23/18
Surgery (yes/no)	9/32
Ablation (yes/no)	5/36
TKI drugs (yes/no)	7/34

ECOG: Eastern Cooperative Oncology Group; BCLC: Barcelona clinical liver classification; TACE: transarterial chemoembolization; TKI: tyrosine kinase inhibitor.

**Table 2:** Clinical information of unresectable secondary liver cancer patients (*n* = 18).

Characteristics	Statistics
Sex (male/female)	12/6
Age (≥65 years old/<65 years old)	10/8
Medical history	
Hepatitis (yes/no)	0/18
Alcohol (yes/no)	1/17
Liver cirrhosis (yes/no)	0/18
Tumor diameter (≥50 mm/<50 mm)	5/13
Tumor number	2.12 ± 1.18
Portal vein invasion (yes/no)	0/18
ECOG (0/1/2)	1/16/1
Child–Pugh(A/B)	18/0
Primary lesion	
Colorectal cancer	8
Gastric cancer	5
Esophagus cancer	1
Pancreatic neuroendocrine tumor	1
Bile duct cancer	1
Lung cancer	2
Treatment history	
Conventional TACE (yes/no)	4/14
Surgery (yes/no)	11/7
Ablation (yes/no)	5/13
TKI drugs (yes/no)	7/11

ECOG: Eastern Cooperative Oncology Group; TACE: transarterial chemoembolization; TKI: tyrosine kinase inhibitor.

**Table 3:** The efficacy for the patients treated with DEB-TACE (mRECIST).

Evaluation	Primary liver cancer (*N*, %)	Secondary liver cancer (*N*, %)
CR	3 (7.32)	0
PR	13 (31.71)	6 (33.33)
SD	21 (51.22)	11 (61.11)
PD	4 (9.76)	1 (5.56)
DCR	90.24	94.44
ORR	39.02	33.33

CR: complete response; PR: partial response; SD: stable disease; PD: progressive disease; DCR: disease control rate; ORR: objective response rate.

**Table 4:** Univariate analysis of factors related to response.

	Primary liver cancer		Secondary liver cancer	
	Nonresponse/response	*P* value	Nonresponse/response	*P* value
Sex				
Male	21/16	0.25	7/3	0.74
Female	4/0		5/3	
Ablation history				
No	21/15	0.35	7/6	0.19
Yes	4/1		5/0	
TKI history				
No	19/15	0.29	7/4	0.73
Yes	6/1		5/2	
c-TACE history				
No	7/11	0.11	8/6	0.28
Yes	18/5		4/0	
Tumor diameter				
<50 mm	9/7	0.87	9/4	0.71
≥50 mm	16/9		3/2	

TKI: tyrosine kinase inhibitor; TACE: transarterial chemoembolization.

## Data Availability

The data used to support the findings of this study are included within.

## Abbreviations

HCC: Hepatocellular carcinoma
c-TACE: Conventional transarterial chemoembolization
DEB-TACE: Drug-eluting bead chemoembolization
CRC: Colorectal cancer
GC: Gastric cancer
OS: Overall survival
DFS: Disease-free survival
PFS: Progression-free survival
AASLD: American Association for the Study of Liver Diseases
ECOG: Eastern Cooperative Oncology Group
DSA: Digital subtraction angiography
ORR: Objective response rate
CR: Complete response
PR: Partial response
DCR: Disease control rate
SD: Stable disease
NRS: Numerical rating scale
CTCAE: Common terminology criteria for adverse events 4.0
ALT: Glutamic-pyruvic transaminase
AST: Glutamic oxalacetic transaminase
TB: Total bilirubin
CEA: Carcinoembryonic antigen
CA-199: Carbohydrate antigen-199.

## Additional Points

(1) DEB-TACE demonstrated 39.02% ORR and 90.24% DCR in primary liver cancer. (2) DEB-TACE showed 33.33% ORR and 94.44% DCR in secondary liver cancer. (3) The treatment-related adverse reactions of DEB-TACE were tolerable.
